# Heterogeneity and Delayed Activation as Hallmarks of Self-Organization and Criticality in Excitable Tissue

**DOI:** 10.3389/fphys.2019.00869

**Published:** 2019-07-05

**Authors:** Andraž Stožer, Rene Markovič, Jurij Dolenšek, Matjaž Perc, Marko Marhl, Marjan Slak Rupnik, Marko Gosak

**Affiliations:** ^1^Faculty of Medicine, University of Maribor, Maribor, Slovenia; ^2^Faculty of Natural Sciences and Mathematics, University of Maribor, Maribor, Slovenia; ^3^Faculty of Education, University of Maribor, Maribor, Slovenia; ^4^Faculty of Energy Technology, University of Maribor, Krško, Slovenia; ^5^Center for Applied Mathematics and Theoretical Physics, University of Maribor, Maribor, Slovenia; ^6^Complexity Science Hub Vienna, Vienna, Austria; ^7^Institute of Physiology and Pharmacology, Medical University of Vienna, Vienna, Austria; ^8^Alma Mater Europaea – ECM, Maribor, Slovenia

**Keywords:** excitable cells, self-organized criticality, beta cells, calcium imaging, computational model, cellular heterogeneity, activation delay

## Abstract

Self-organized critical dynamics is assumed to be an attractive mode of functioning for several real-life systems and entails an emergent activity in which the extent of observables follows a power-law distribution. The hallmarks of criticality have recently been observed in a plethora of biological systems, including beta cell populations within pancreatic islets of Langerhans. In the present study, we systematically explored the mechanisms that drive the critical and supercritical behavior in networks of coupled beta cells under different circumstances by means of experimental and computational approaches. Experimentally, we employed high-speed functional multicellular calcium imaging of fluorescently labeled acute mouse pancreas tissue slices to record calcium signals in a large number of beta cells simultaneously, and with a high spatiotemporal resolution. Our experimental results revealed that the cellular responses to stimulation with glucose are biphasic and glucose-dependent. Under physiological as well as under supraphysiological levels of stimulation, an initial activation phase was followed by a supercritical plateau phase with a high number of global intercellular calcium waves. However, the activation phase displayed fingerprints of critical behavior under lower stimulation levels, with a progressive recruitment of cells and a power-law distribution of calcium wave sizes. On the other hand, the activation phase provoked by pathophysiologically high glucose concentrations, differed considerably and was more rapid, less continuous, and supercritical. To gain a deeper insight into the experimentally observed complex dynamical patterns, we built up a phenomenological model of coupled excitable cells and explored empirically the model’s necessities that ensured a good overlap between computational and experimental results. It turned out that such a good agreement between experimental and computational findings was attained when both heterogeneous and stimulus-dependent time lags, variability in excitability levels, as well as a heterogeneous cell-cell coupling were included into the model. Most importantly, since our phenomenological approach involved only a few parameters, it naturally lends itself not only for determining key mechanisms of self-organized criticality at the tissue level, but also points out various features for comprehensive and realistic modeling of different excitable systems in nature.

## Introduction

Self-organized collective dynamics is a remarkable phenomenon observed in various natural and man-made systems, in which collective behavior emerges from local interactions between individual elements (Bak, 1996; Marković and Gros, 2014; Ellis and Kopel, 2019). Regardless of the specific mechanisms responsible for self-organization, the resulting coherent global structures or dynamics are characterized by scale-invariant properties and a power-law distribution of systems’ observables (Khaluf et al., 2017; Muñoz, 2018). Such emergent behavior is often associated with critical dynamics and is assumed to be particularly beneficial for the functioning of several living systems, from the microscopic to the macroscopic (Tamayo et al., 1999; Nykter et al., 2008; Chialvo, 2010; Bialek et al., 2012; Furusawa and Kaneko, 2012; Sasai, 2013; Allegrini et al., 2015; Muñoz, 2018). Criticality has been argued to originate from the fact that many biological systems operate in the vicinity of a critical point of a phase transition between an ordered and disordered phase, which ensures a balance between robustness against perturbations and flexibility to adapt to a changing environment. However, the exact reasons why signatures of criticality can be conjectured to emerge in living systems are still under debate and the underlying principles are incompletely understood (Lovecchio et al., 2012; Moretti and Muñoz, 2013; Nonnenmacher et al., 2017). Most importantly, despite some skepticism and limitations, the evidently increasing amount of empirical evidence, fostered also by technological and computational advances, is nowadays inspiring more and more researchers to investigate the complexity of biological systems through the lens of phase transition behavior and criticality.

In the domain of biological networks, the concepts of self-organization and criticality have received the most attention in the field of neuroscience. On the smallest scales, patterns of activity in neuronal populations have been found to be very heterogeneous, with sizes of so-called neuronal avalanches following a power law distribution (Beggs and Plenz, 2003; Pasquale et al., 2008; Timme et al., 2016). Empirical evidence for criticality has been reported in both different *in vivo* preparations and on larges scales of whole-brain imaging (Plenz and Thiagarajan, 2007; Haimovici et al., 2013; Hesse and Gross, 2014). The presence of emergent critical dynamics in the nervous system is theoretically appealing and consequently computational models and tools from the realms of statistical physics have been utilized to unveil the mechanisms and correlations between phase transition behavior and the occurrence of scale-invariant neuronal avalanches (Plenz and Thiagarajan, 2007; Rubinov et al., 2011; Friedman et al., 2012; Zare and Grigolini, 2013; Tkačik et al., 2015; Brochini et al., 2016; di Santo et al., 2018). Importantly, several studies have underscored the emergence of critical dynamics in neuronal networks as one of the key pillars for their optimal operational abilities (Kinouchi and Copelli, 2006; Shew and Plenz, 2013; Shew et al., 2015; Stoop and Gomez, 2016). Moreover, complex and hierarchically organized network structures along with neuronal plasticity were identified as the main neurophysiological determinants that ensure robust critical behavior (Levina et al., 2007; Rubinov et al., 2011; Moretti and Muñoz, 2013; Hutt et al., 2014; Massobrio et al., 2015b). It should be noted that deviations from critical behavior occur in neuronal networks during development (Tetzlaff et al., 2010; Massobrio et al., 2015b) and under pathological conditions (Massobrio et al., 2015a; Tagliazucchi et al., 2016; Hahn et al., 2017). Especially during epileptic seizures (Hobbs et al., 2010; Meisel et al., 2012) or by pharmacological disruptions of the excitation-inhibition balance (Barral and D Reyes, 2016), an excess of large system-spanning avalanches occur, as is characteristic for supercritical dynamical states. Consequently, it has been hypothesized that the healthy brain resides near a critical or even slightly subcritical state, thereby ensuring a safety margin from supercriticality, which has been linked to some pathophysiological disorders (Priesemann et al., 2014; Tomen et al., 2014; Massobrio et al., 2015a).

Notably, recent research indicates that the concept of critical dynamics and power-law scaling in living beings applies well beyond the spatiotemporal activity patterns of neurons. At the (sub)cellular level, mitochondrial network of heart myocytes was reported to operate at the edge of dynamic instability characterized by a fractal scaling of depolarized mitochondrial clusters (Aon et al., 2004). In this regime, constancy in terms of a steady supply of ATP is provided in combination with flexibility, which ensures the adaptation of energy production in accordance with metabolic demands (Aon et al., 2006). Moreover, hallmarks of self-organized criticality have also been observed in the spatiotemporal organization of Ca^2+^ waves. Jung et al. (1998) reported a power law distribution of noise-induced spiral Ca^2+^ wave sizes in cultured networks of astrocytes. Heavy-tailed distributions and an avalanche-like behavior have also been observed in intracellular Ca^2+^ signalization in cardiac myocytes (Nivala et al., 2012) and in immature oocytes (Lopez et al., 2012). In both studies Ca^2+^ waves in individual cells resulted from random local Ca^2+^ events, reflecting small Ca^2+^ release events from individual channels or a cluster of channels, which can occasionally integrate to global events reflecting a whole-cell Ca^2+^ signal (Berridge et al., 2000). As the localized subcellular Ca^2+^ events interact, e.g., via diffusion, they can self-organize and lead to avalanches of activity that propagate through the cell. The concept does not only assert that the extent of such events is characterized by scale invariance, but also makes the global Ca^2+^ signals appear rather deterministic in spite of their stochastic origin (Skupin et al., 2008).

Even though information processing in living organisms is often performed by large networks of interacting cells, little attention has been devoted to the principles underlying critical dynamics on multicellular and tissue levels of organization. Recently, we have empirically shown that fingerprints of criticality are also found in the spatiotemporal dynamics of interconnected beta cells from islets of Langerhans (Gosak et al., 2017). These endocrine cells synthesize and release insulin, the anabolic hormone which promotes postprandial storage of nutrients, and thus serves a crucial role in homeostasis of energy that becomes disrupted in diabesity (Kahn et al., 2014). Insulin concentration in the blood displays inherent multimodal oscillations (Satin et al., 2015), and several studies have attempted to reveal the underlying mechanism by providing links to oscillations in beta cells metabolism and to a feedback between ion channels and electrical activity. This was further corroborated by modeling the interplay of the two signaling aspects (Bertram et al., 2007). Moreover, recent theoretical studies emphasized the role of biphasic feedback circuits in controlling functional beta cell mass (Karin et al., 2016) and progression of diabetes mellitus (Karin and Alon, 2017). On the organizational level of a single islet, beta cells respond to nutrient stimulation with an initial transient depolarization, followed by fast oscillations in membrane potential that are superimposed on a plateau phase (Gilon and Henquin, 1992; Rorsman and Braun, 2013; Skelin Klemen et al., 2017). Intracellular Ca^2+^ concentration ([Ca^2+^]_*IC*_) closely follows changes in membrane potential due to tight coupling between electrical and calcium dynamics in beta cells (Gilon and Henquin, 1992; Dolenšek et al., 2013). However, all beta cells within an islet do not show identical electrical or [Ca^2+^]_*IC*_ activity and can therefore not be regarded as uniformly and strongly coupled identical units or even as a single supercell. Rather, the collective activity of beta cells is characterized by a phase shift between individual cellular oscillations, ultimately resulting in heterogeneous waves of membrane potential and [Ca^2+^]_*IC*_ changes. These waves spread repetitively over an islet, but not always from the same source and not always throughout the whole syncytium (Benninger et al., 2008; Dolenšek et al., 2013; Stožer et al., 2013a). They are thought to originate in specific sub-regions with elevated excitability (Benninger et al., 2014) or higher intrinsic oscillation frequency (Westacott et al., 2017). A plethora of evidence demonstrates that an essential prerequisite for the coordinated beta cell activity and formation of waves is intact intercellular connectivity mediated via gap junctions (Calabrese et al., 2003; Ravier et al., 2005; Bavamian et al., 2007; Bosco et al., 2011; Benninger et al., 2014) and probably other modes of communication, such as paracrine, contact-dependent, and ciliary signaling (Squires et al., 2002; Konstantinova et al., 2007; Yang et al., 2011; Gerdes et al., 2014). Most importantly, intercellular connectivity is not only necessary for normal islet function, its perturbations were also linked to metabolic diseases and impaired insulin secretion (Hamelin et al., 2009; Carvalho et al., 2012; Head et al., 2012; Hodson et al., 2013; Benninger and Piston, 2014; Benninger et al., 2018; Nasteska and Hodson, 2018).

Moreover, individual beta cells are intrinsically highly heterogeneous (Gutierrez et al., 2017). Several different approaches have demonstrated relatively large differences in the extent of coupling between beta cells (Pérez-Armendariz et al., 1991; Farnsworth et al., 2014), as well as different levels of excitability (Jonkers and Henquin, 2001; Benninger et al., 2011) and rates of glucose metabolism (Benninger et al., 2014; Benninger and Hodson, 2018; Nasteska and Hodson, 2018). Because of these features, the spatiotemporal responses of beta cells are very complex and to understand how a population of these heterogeneous and heterogeneously coupled cells activate to work in synchrony is a hot topic in islet physiology research (Pedersen et al., 2013; Benninger and Piston, 2014; Benninger et al., 2014; Markovič et al., 2015; Cappon and Pedersen, 2016). Motivated by complexity science approaches, several studies have investigated beta cell responses in terms of phase transition behavior (Hraha et al., 2014; Loppini et al., 2014; Stamper et al., 2014). In this vein, in our recent study we demonstrated that under physiological circumstances, the initial response to glucose is characterized by a power-law probability distribution of Ca^2+^ wave sizes, which can be maintained in the long run by periodic stimulation, but changes to supercriticality upon constant stimulation, thereby demonstrating empirically the fingerprints and basic preconditions of critical behavior (Gosak et al., 2017).

In the present work, we extend our preceding research to supraphysiological glucose concentrations that are usually used in experiments, but accompany pathophysiological states *in vivo*. It turns out that higher glucose levels evoke more rapid and qualitatively different beta cell responses when compared to physiological levels of stimulation. To assess the measured non-trivial and rich dynamical patterns, we propose a phenomenological model of coupled excitable cells that accounts for the observed physiological as well as pathophysiological behavior and encompasses both beta cell signaling specifics and heterogeneity. Moreover, in contrast to the exhaustive computational models with many parameters, our phenomenological modeling approach made it easier to empirically explore the necessary ingredients and physiological determinants that ensure a good overlap between experimental and computational results.

## Materials and Methods

### Multicellular Calcium Imaging in Pancreatic Tissue Slices

Acute pancreatic tissue slices were prepared as described previously (Speier and Rupnik, 2003; Stožer et al., 2013a). Briefly, low-melting point agarose (1.9% V/V) was injected into the proximal common bile duct that was clamped distally at the major duodenal papilla. Retrograde inflow of the agarose served, once cooled, as a scaffold for subsequent tissue cutting into 140 μm thick slices on a vibratome (VT 1000 S, Leica). Staining with the calcium sensitive dye Oregon Green 488 BAPTA-1 AM [6 μM final concentration, 0.03% Pluronic F-127 (w/v), and 0.12% dimethylsulphoxide (v/v) dissolved in HEPES-buffered saline at RT, consisting of (in mM) 150 NaCl, 10 HEPES, 6 glucose, 5 KCl, 2 CaCl_2_, 1 MgCl_2_; titrated to pH = 7.4 using 1 M NaOH], was performed for 50 min at RT. Confocal imaging of calcium dynamics was performed on the Leica TCS SP5 AOBS Tandem II upright confocal system (20x HCX APO L water immersion objective, NA 1.0) and Leica TCS SP5 DMI6000 CS inverted confocal system (20X HC PL APO water/oil immersion objective, NA 0.7), utilizing a perifusion system filled with extracellular solution, consisting of (in mM) 125 NaCl, 26 NaHCO_3_, 6 lactic acid, 3 myo-inositol, 2.5 KCl, 2 Na-pyruvate, 2 CaCl_2_, 1.25 NaH_2_PO_4_, 1 MgCl_2_, 0.5 ascorbic acid and added either substimulatory 6 mM or stimulatory 8 or 12 mM glucose. The calcium dye was excited at 488 nm via an argon laser line and the emitted fluoresces was detected in the range of 500–700 nm by a Leica HyD detector. Time series were acquired at 10 Hz (512 × 512 pixels). Further analysis was done off-line by manually selecting ROIs corresponding to beta cells. The exported time series of the F/F_0_ ratio were then further processed as explained in the continuation.

### Computational Model

#### Phenomenological Single Cell Model

We utilized a phenomenological model to describe the dynamics of the electrically excitable beta cells. In particular, we made use of a two-dimensional iterated map proposed by Rulkov (2002):

(1) $$u_{i}\,\left(t+1\right)=\alpha_{i}\,\left(t\right)\,/\,\left(1+u_{i}\,{\left(t\right)}^{2}\right)+v_{i}\,\left(t\right)+g_{i}\,\sum_{j}\varepsilon_{i\,j}\,\left(u_{j}-u_{i}\right)+\beta \,\xi_{i}\,\left(t\right),$$

(2) $$v_{i}\,\left(t+1\right)=v_{i}\,\left(t\right)-\sigma \,u_{i}\,\left(t\right)-\chi ,$$

where *u*_i_(*t*) and *v*_i_(*t*) are the slow and the fast dynamical variables for the *i*-th cell, respectively, and are considered as dimensionless variables, *t* is the discrete time index, α_i_, χ, and σ are systems parameters, and β = 0.0045 defines the strength of Gaussian noise with zero mean and unit variance that accounts for stochasticity in beta cell dynamics. The fast variable *u*_i_(*t*) describes the dynamics of the membrane potential of the cell, whereas the slow variable *v*_i_(*t*) reflects the gating variable. Although this is an abstract and simple mathematical model, it mimics well the basic principles of more complex cellular behaviors that are observed in different cell types, including beta cells.

More specifically, in terms of metabolic changes, electrical activity, [Ca^2+^]_*IC*_ dynamics, and insulin secretion, beta cells within islets respond to stimulation by glucose in two phases. During the first phase which is transient, they show elevated levels of intracellular ATP and NAD(P)H, followed electrically by very fast bursting or continuous bursting. Bursts are periods of very fast depolarizations called spikes that last a few seconds and continuous bursting consists of an uninterrupted set of spikes at a frequency around 10 Hz. At the level of [Ca^2+^]_*IC*_ dynamics, this first phase consists of a transient increase in [Ca^2+^]_*IC*_, during the ascending part of which a few fast [Ca^2+^]_*IC*_ oscillations may be present, reflecting fast burst before continuous bursting. It should be pointed out that at present the very fast spikes cannot be resolved in [Ca^2+^]_*IC*_ imaging. This first phase lasts a few minutes and at the level of hormone output overlaps with the first phase of insulin secretion. During the second phase, continuous bursting and the accompanying transient increase in [Ca^2+^]_*IC*_ change to regular bursting and corresponding fast [Ca^2+^]_*IC*_ oscillations. At the level of metabolism and hormone secretion, NADPH and ATP are elevated during this period and insulin secretion shows a stable second phase (Henquin and Meissner, 1984; Gilon and Henquin, 1992; Li et al., 2013, 2014; Gilon et al., 2014; Skelin Klemen et al., 2017; Rorsman and Ashcroft, 2018). It should be noted that during this second phase, insulin is also secreted in bursts synchronized with electrical bursts and fast [Ca^2+^]_*IC*_ oscillations (Gilon et al., 1993; Bergsten et al., 1994). This fast electrical, [Ca^2+^]_*IC*_, and secretory pattern is superimposed on a slower set of oscillations in ATP, membrane potential, [Ca^2+^]_*IC*_, insulin secretion, and some other parameters, which has been reviewed in detailed elsewhere (Satin et al., 2015). A further layer of complexity to this behavior comes from the fact that it is glucose-dependent. In higher glucose, the frequency or duration of bursts and correspondingly the fast [Ca^2+^]_*IC*_ oscillations increase, such that the active time and insulin secretion increase. Noteworthy, it seems that the underlying slow pattern is not glucose-dependent (Satin et al., 2015; Gosak et al., 2017; Skelin Klemen et al., 2017).

Most importantly, modeling all of the above aspects of beta cell responses to glucose requires the use of realistic biophysical models. However, in this study we focused only on fast [Ca^2+^]_*IC*_ dynamics which can be satisfactorily captured by the phenomenological model employed here. Most importantly, since we wanted to study the effects of various types of heterogeneities in a network of coupled beta cells, in comparison with a genuine biophysical model, a phenomenological description of the complex cellular dynamics is not only beneficial in terms of numerical efficiency, but also enables exploration of the system with very few free parameters.

**FIGURE 1:**
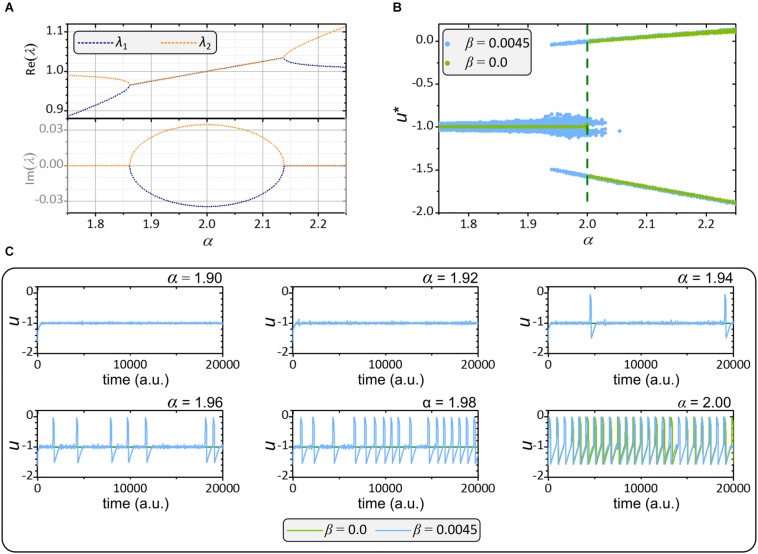
Dynamical features of the Rulkov map. **(A)** Real and imaginary eigenvalues λ_1_ and λ_2_ for different values of the control parameter α. **(B)** Bifurcation diagram of the fast variable with (blue) and without (green) added noise. **(C)** Traces of the fast variable with (blue) and without (green) noise for different values of excitability levels α.

The Rulkov map displays a variety of dynamics depending on the parameter choice, as extensively investigated in the past (Rulkov, 2002; Ibarz et al., 2011; Markovič et al., 2012). To better understand the dynamical phases that occur in our study, we performed a stability analysis. For χ = σ the fixed point equals u^*^ = −1 and v^*^ = −1 − α/2. If α < 2 the steady state is stable and for α < 1.86 the fixed point is asymptotically stable, since the both eigenvalues, λ_*1*_ and λ_*2*_ have only real parts and their absolute value is less than 1 (see Figure 1A). For the values 1.86 < α < 2, the fixed point is still stable (|*Re*(λ_1_)| < 1 and |*Re*(λ_2_)| < 1), but the eigenvalues are complex making the fixed point a spiral sink. For the values of bifurcation parameter 2 < α ≤ 4, the solution (u^*^,v^*^) becomes unstable and the system exhibits sustained periodic pulses, chaotic bursts of pulses and sustained chaotic pulsing. In our study we focused on the region 1.86 < α < 2, where the steady state is excitable and oscillations can be induced by noise and/or heterogeneity. This is shown in the bifurcation diagram in Figure 1B. For the chosen noise level, oscillations occur at α > 1.93. Noteworthy, with increasing α the excitability level and the cellular activity increase as well. Therefore, increasing α in our model emulates the decrease in glucose-induced K_*ATP*_-channel conductance, the main trigger of beta cells in realistic models (Stamper and Wang, 2019). The temporal behavior of our single-cell phenomenological model is visualized in Figure 1C, for different values the bifurcation parameter *α*.

#### Intercellular Coupling Model

**FIGURE 2:**
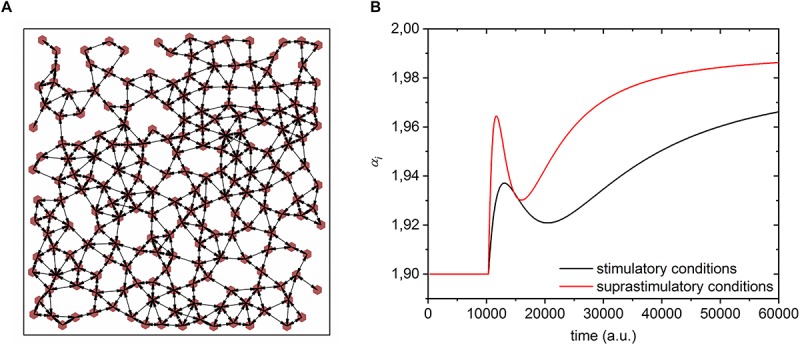
Features of the phenomenological model of beta cell population. **(A)** A typical simulated beta cell network architecture. Red dots denote individual cells and the arrows depict intercellular electrical coupling. **(B)** Simulated time course of beta cell excitability rate after switching from substimulatory to stimulatory (Δα = 0.08, *A* = 0.45, *B* = 0.0004, $T_{m,i}^{\left(8\right)}=35000$, *t*_*s*_ = 10000) and suprastimulatory (Δα = 0.09, *A* = 0.70, *B* = 0.0008, $T_{m,i}^{\left(12\right)}=20000$, *t*_*s*_ = 10000) conditions.

The sum in Eq. (1) signifies the electrical coupling and it runs over all cells, whereby ε_*ij*_ = 1 if the unit *i* is coupled to unit *j*, whilst otherwise ε_*ij*_ = 0. *g*_*i*_ is the coupling constant. The structure of the intercellular coupling between beta cells was modeled by the random geometric graph model (Penrose, 2007). First, all *N=200* cells were arranged randomly in a unit square with a prescribed minimal possible distance (0.04) to ensure a more homogeneous and realistic spatial distribution of cells. Then, the *i*-th and the *j*-th cell were considered to be connected, i.e., ε_*ij*_ = 1, if their physical distance was less than $r_{i\,j}=\sqrt{\left\langle k\right\rangle /\left(N\,\pi \right)}$, where ⟨*k*⟩ = 6 signifies the average number of connections per cell. A typical intercellular network structure is shown in Figure 2A.

#### Modeling the Temporal Responses to Stimulation

To simulate the progressive recruitment of beta cells after switching from substimulatory to stimulatory or suprastimulatory glucose concentrations, we introduced a time-dependent function for the parameter α_*i*_ that reflects the cellular excitability level:

(3) $$\alpha_{i}\,\left(t\right)=\alpha_{0}+\Delta \,\alpha_{i}\,\left[A\,\left(t-t_{s}\right)\,B\,e^{-B\,\left(t-t_{s}\right)+1}+\frac{{\left(t-t_{s}\right)}^{2}}{{\left(t-t_{s}\right)}^{2}+{\left(T_{m,i}-t_{s}\right)}^{2}}\right],$$

In this manner, we took into account the delay due to glucose metabolism. In Eq. (3) α_0_ = 1.90 is the basal substimulatory level of excitability with no activity, Δα is the amplitude of the increased excitability for the *i*-th cell provoked by increased glucose concentration, and *t*_*s*_ is the initial time before the cells respond to stimulation. The first term within the brackets on the right side of Eq. (3) stands for the initial and the second term for the successive beta cell activations. We implemented such a biphasic and glucose-dependent response to account for the previously observed biphasic and glucose-dependent behavior of beta cells in terms of their metabolic, electrical, [Ca^2+^]_*IC*_, and secretory response described above. The parameters *A* ∈ [0,1] and *B* signify the glucose-dependent amplitude and decay rate of the first activation. The parameter *T*_*m,i*_ specifies the temporal scale of the final activation, i.e., elevation in the level of excitability. On the basis of experimental results we hypothesized that under lower and physiological stimulatory conditions the amplitude of the first response and the decay rate are lower (*A* = 0.45 and *B* = 0.0004) than under high and supraphysiological stimulatory levels (*A* = 0.7 and *B* = 0.0008). Moreover, to account for the longer activation phase observed under 8 mM glucose in comparison to 12 mM stimulation, we set the half-activation times to $T_{m,i}^{\left(8\right)}=35000$ and $T_{m,i}^{\left(12\right)}=20000$. Finally, to resemble a higher intrinsic beta cell activity under higher stimulation, we set the parameters when emulating the behavior under 8 mM glucose to Δα = 0.08 and σ = χ = 0.001 and to Δα = 0.09 and σ = χ = 0.0012 when emulating the behavior under 12 mM glucose. It should be noted that these small changes in the parameters σand χ have an insignificant effect on bifurcation behavior reported in Figure 1. Temporal traces of simulated excitability rates when switching to stimulatory and suprastimulatory conditions are shown in Figure 2B. Since the parameter α regulates the cellular activity (see Figure 1), by this means a stimulation-specific temporal recruitment of beta cells is modeled.

#### Heterogeneity of Beta Cells

**FIGURE 3:**
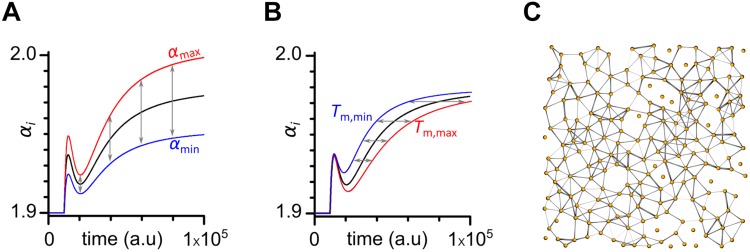
A schematic representation of all three types of heterogeneities in the model. **(A)** electrical excitability Δα_*i*_, **(B)** beta cell metabolism *T*_m,*i*_, **(C)** electrical coupling *g*_*i*_. Widths of connections reflect the coupling strength.

Previous studies have suggested an extensive heterogeneity among β cells due to differences in topography, cell sizes, functional maturity, channel densities, intercellular coupling, rates of glucose metabolism, membrane potential changes, [Ca^2+^]_*IC*_ oscillations, granule content, and secretory capacity, to name only a few examples (MacDonald and Rorsman, 2006; Benninger and Piston, 2014; Bader et al., 2016; Roscioni et al., 2016; Pipeleers et al., 2017; Skelin Klemen et al., 2017; Benninger and Hodson, 2018; Nasteska and Hodson, 2018). To robustly account for the abovementioned differences in glucose sensitivity and metabolism, electrical excitability and [Ca^2+^]_*IC*_ signals, as well as intercellular coupling strength, we introduced in our phenomenological model heterogeneity three crucial aspects of cellular signaling: (i) stimulation-induced temporal change in excitability (parameter *T*_m,*i*_), (ii)stimulation-dependent increase in excitability (parameter Δα_*i*_), and (iii) intercellular coupling strength (parameter *g*_*i*_). All three parameters were assumed to follow a truncated normal distribution with a relative standard deviation of 30% and a cut-off of 90%. The three types of cellular heterogeneity are schematically visualized in Figure 3.

### Processing of Time Series and Activity Pattern Classification

Time series of individual cells obtained from experimental recordings were first accordingly processed to achieve a coherent and accurate binarization. The main task of this pre-processing step is to level and smooth the individual time series, remove noise, and firmly extract the fast component of Ca^2+^ oscillations. To this purpose, we utilized a band pass filter, whereby the frequency band of interest was determined by visual assessment. The filtered signal was then additionally smoothed with standard sliding window algorithm, with a window size of four frames (Yaroslavsky et al., 2001). Preprocessing of computationally obtained traces was not required. In continuation the time series from experiments and simulations will be referred to as *x* and the corresponding value at (discrete) time *t* as *x*(*t*). The following binarization procedure was based on the: (i) standard deviation *std*[*x*], (ii) first derivative of time series *x*′ (*t*), and (iii) standard deviations of its the first derivative *std* [*x*′]. First, we have defined the potential onset and ending times of individual Ca^2+^ spikes by searching for local extremes in the first derivative. More precisely, our algorithm searches for local maxima’s, which satisfy the condition *x*′ (*t*) > 1.5*std* [*x*′]. The time, at which the local maxima is found is then the potential onset of Ca^2+^ spikes, *t*_*START*_. In a time forward direction, we than seek the first local minima that satisfies the condition *x*′ (*t*) < −1.5*std* [*x*′] and store its time of occurrence, *t*_*END*_. Lastly we test, if the local maximum of the Ca^2+^ signal within the time interval *t* ∈ [*t*_*START*_,*t*_*END*_] satisfies the condition *x*(*t*) > 1.5*std* [*x*]. The corresponding binary time series takes on a value of 1 in all time intervals *t* ∈ [*t*_*START*_,*t*_*END*_], where the three conditions are satisfied, whilst otherwise the value is 0.

Afterward, we used binarized time series and the physical positions of individual cells to merge spatially and temporally synchronized events into clusters by performing the space-time cluster analysis, as proposed by Jung (1997) and Jung et al. (1998). In brief, we combined the information about the positions of cells and their binary traces into a space-time cube (STC). In this STC we defined a cubic region of interest (STC-ROI) in which we search for active cells. If two cells in neighboring STC were simultaneously active, they were considered to belong to the same cluster. In other words, we traced the course of the wave from cell-to-cell and if the nearby cells became activated within a short time period and if they were close enough, the given activation was considered as one individual cluster with size *p*. By this means, an individual STC contains the information about the number of cells that were activated in a given excitation wave, as well as about the temporal extent of the given event, as described previously (Gosak et al., 2017). The spatial side-length of the STC was determined as the average distance to 6 closest neighbors (∼25 μm in experiments and ∼0.12 in simulations). The temporal side-length was determined empirically, so that a firm of discrimination of individual waves was attained. To quantify the spatiotemporal activity patterns in experiments and simulations, we calculated the distribution of cluster sizes *N*(*p*) for different stimulation protocols and activity phases. Finally, the results were fitted with a power-law function to qualitatively evaluate the nature of the distribution, i.e., critical vs. supercritical behavior. In particular, by visually assessing deviations from the power-law distribution in the form of an excess of global events we determine the supercritical nature of the spatio-temporal activity, whereas a close-to-power-law behavior implies critical-like behavior, as suggested previously (Levina et al., 2007; Friedman et al., 2012).

## Results

First, we present experimentally measured beta cells activity after switching from substimulatory to stimulatory and suprastimulatory levels of glucose. Then, we show the results of our computational model of interconnected excitable cells, which was designed to mimic the activity patterns observed in experiments under physiological as well as under supraphysiological levels of stimulation. Data either from experiments or from simulations were handled in the same manner, in order to provide foundation for further characterization of the spatiotemporal activity.

### Experimental Results

**FIGURE 4:**
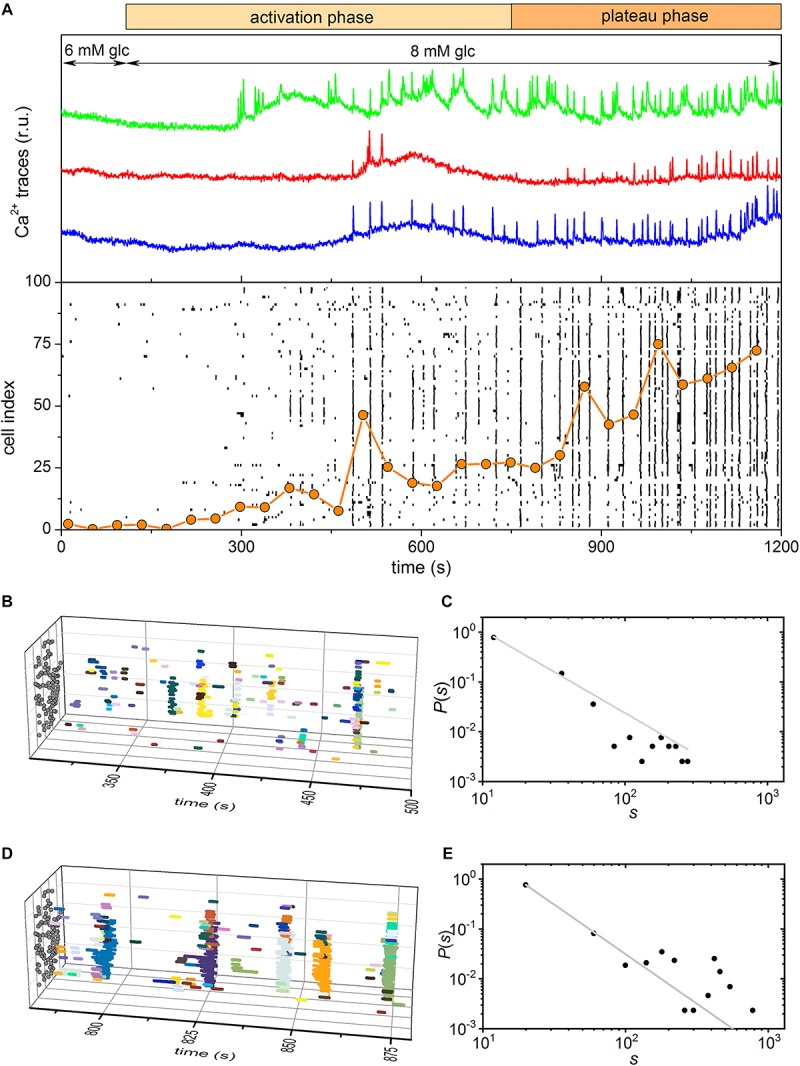
Experimentally measured beta cell responses after stimulation with 8 mM glucose. **(A)** Three characteristic Ca^2+^ traces and the raster plot of binarized Ca^2+^ activity of all cells in the islet. The orange dotted line indicates the fraction of active cells within the given time-window that was slid throughout the recording. **(B,D)** 3D raster plots showing the Ca^2+^ activity waveforms for selected intervals for the activation **(B)** and plateau **(D)** phase. Colors denote specific Ca^2+^ events. Gray dots on the *y*, *z* plane stand for coordinates of cells. **(C,E)** The distributions of Ca^2+^ wave sizes for the activation **(C)** and plateau **(E)** phase. The gray dashed line indicates the power-law fit. The slope in the critical-like activation phase is –1.69.

**FIGURE 5:**
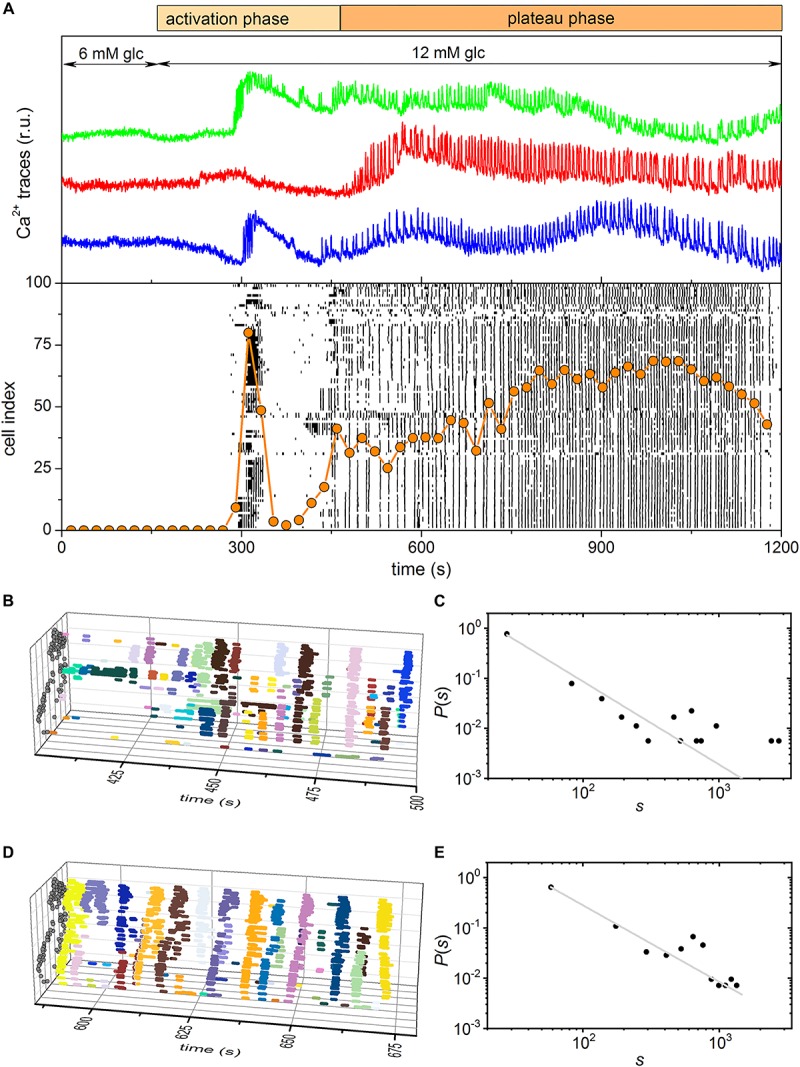
Experimentally measured beta cell responses after stimulation with 12 mM glucose. **(A)** Three characteristic Ca^2+^ traces and the raster plot of binarized signals of Ca^2+^ oscillations in all cells in the islet. The orange dotted line indicates the fraction of active cells within the given time-window that was slid throughout the recording. **(B,D)** 3D raster plots showing the Ca^2+^ waves for selected intervals for the activation **(B)** and plateau **(D)** phase. Colors denote specific Ca^2+^ events. Gray dots on the *y*, *z* plane stand for coordinates of cells. **(C,E)** The distributions of Ca^2+^ wave sizes for the activation **(C)** and plateau **(E)** phase. The gray dashed line indicates the power-law fit.

To record beta cell responses to glucose stimulation, we used multicellular confocal imaging on acute tissue slices as described in Materials and methods. We stimulated islets with two glucose concentrations: one commonly observed *in vivo*, i.e., 8 mmol/l, and one measured in conditions of stress or glucose intolerance, i.e., 12 mmol/l. We termed the former physiological and the later supraphysiological concentration. Following either stimulus, beta cells exhibited a two-phase response: (i) an activation phase, characterized by a transient increase in [Ca^2+^]_*IC*_ and presence of fast oscillations, during which beta cells were gradually recruited, and (ii) a subsequent plateau phase, characterized by repeated and more regular oscillations of now fully recruited beta cells (Figures 4A, 5A). Heterogeneity of beta cells responses was reflected in the time window during which cells activate within an islet. These intervals differ for the two stimulatory concentrations: about 600 s (100 s < t < 700 s, Figure 4A) for the physiological and about 300 s (150 s < t < 450 s, Figure 5A) for the supraphysiological concentration. To surpass the qualitative description of the two phases, we looked for collective spatiotemporal behavior of beta cells. To this aim, we meticulously detected Ca^2+^ waves during both phases, and plotted them as individual events in space-time for better visualization. While being stimulated with the physiological concentration, the activation phase exhibited very heterogeneous spatiotemporal behavior, one that resulted in calcium waves of very different sizes (Figure 4B). The following plateau phase evoked a more regular pattern of [Ca^2+^]_*IC*_ oscillations, with prevailing global intercellular calcium waves that encompassed often the majority of the cells within an islet (Figure 4D). However, the activation/plateau pattern changed during supraphysiological stimulation. Majority of the cells responded with a rapid burst of oscillatory activity followed by brief refractory period during the activation phase (Figure 5B). The subsequent plateau phase was dominated by global intercellular calcium waves (Figure 5D). To be able to quantify the former description, we determined the distribution *P*(*s*) of relative wave sizes *s* and plotted it on log-log scale (Figures 4C,E, 5C,E). While comparing *P*(*s*) for the two concentrations in question, we observed that in the lower concentration the *P*(*s*) in the activation phase followed the power law, whereas the plateau phase was again dominated by global waves. Such switching in behavior from the critical to the supercritical was not observed in the higher stimulatory concentration, during which the behavior was locked to the supercritical during both phases. Namely, the activation phase under supraphysiological concentrations was too rapid, exhibited a huge activation burst, and lacked the progressive recruitment of cells that featured an emergent behavior with very heterogeneous Ca^2+^ waves.

### Computational Results

**FIGURE 6:**
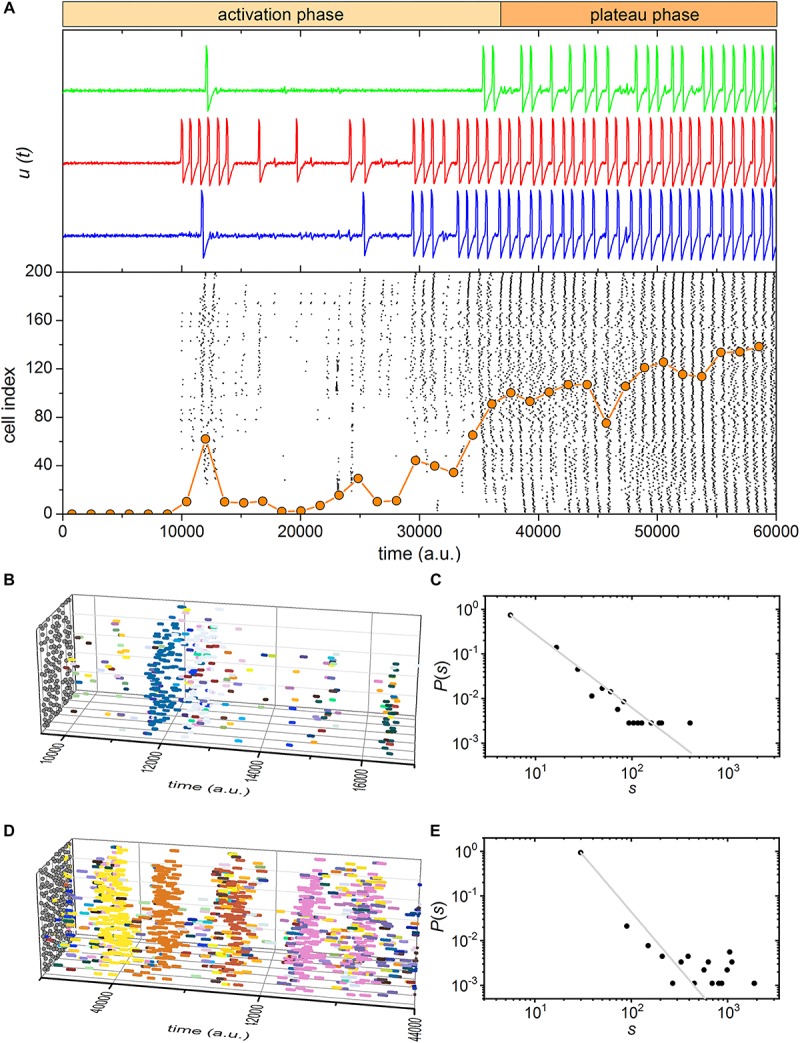
Simulated beta cell responses after switching from a substimulatory to stimulatory levels of stimulation, i.e., from 6 to 8 mM glucose. **(A)** Three characteristic traces of simulated cellular dynamics and the raster plot of binarized cellular activity. The orange dotted line indicates the fraction of active cells within the given time-window that was slid throughout the simulation. **(B,D)** 3D raster plots showing the excitation waves for selected intervals for the activation **(B)** and plateau **(D)** phase. Colors denote individual waves. **(C,E)** The distributions of excitation wave sizes for the activation **(C)** and plateau **(E)** phase. The gray dashed line indicates the power-law fit. The slope in the critical-like activation phase is –1.64.

**FIGURE 7:**
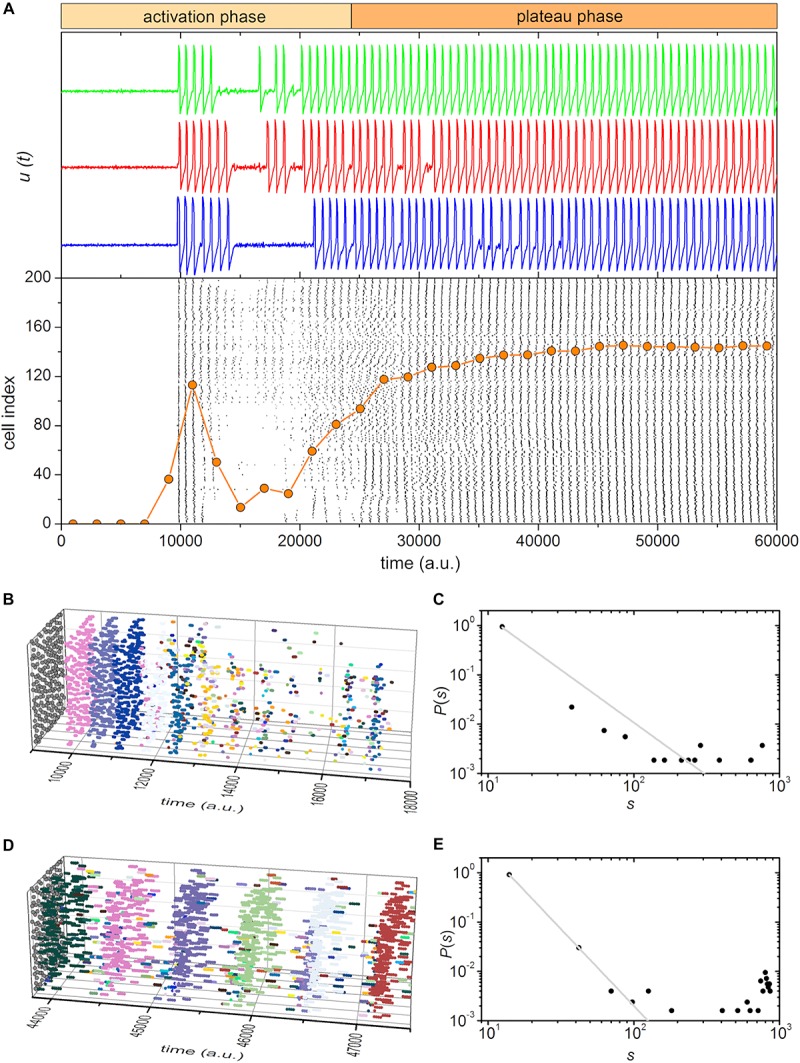
Simulated beta cell responses after switching from a substimulatory to a suprastimulatory level of stimulation, i.e., from 6 to 12 mM glucose. **(A)** Three characteristic traces of simulated cellular dynamics and the raster plot of binarized cellular activity. The orange dotted line indicates the fraction of active cells within the given time-window that was slid throughout the simulation. **(B,D)** 3D raster plots showing the excitation waves for selected intervals for the activation **(B)** and plateau **(D)** phase. Colors denote individual waves. **(C,E)** The distributions of excitation wave sizes for the activation **(C)** and plateau **(E)** phase. The gray dashed line indicates the power-law fit.

We developed a phenomenological model of coupled excitable cells with the aim to explore the prerequisites and mechanisms that lead to complex dynamical behavior observed in experiments. The minimalistic map-based description of excitable dynamics mimics the activity of beta cells. The stimulation was modeled as a heterogeneous and delayed increase in the excitability level, whereby a higher increase was used when supraphysiological stimulation was simulated (see section “Materials and Methods”). To further account for beta cell heterogeneity, we additionally included cell-to-cell variability in the absolute levels of excitability and in the intercellular coupling strength. In this case, we obtained a good qualitative agreement with experimental findings. The results are presented in Figures 6, 7 for the simulation of physiological and supraphysiological stimulations, respectively.

Regardless of the stimulation level, we observed a biphasic response. Most importantly, the activation phase under lower stimulation levels was rather long and exhibited waves of various sizes and many of them were confined to small sub-regions of the islet. This is a result of very heterogeneous responses to stimulation, which in turn led to non-trivial and self-organized dynamical patterns. As in experimental measurement, the distribution of spatiotemporal cluster sizes was found to roughly follow a power-law (Figure 6C), which pinpoints toward a transient phase of critical dynamics. In contrast, the activation phase under supraphysiological conditions was shorter and the wave sizes were larger and more homogeneous, similarly as in the experiment. Consequently, the distribution deviates from the pure power-law behavior, mostly on account of an excess of larger excitation events (Figure 7C). The second plateau phase was qualitatively very similar in both scenarios. In both cases the spatiotemporal activity was dominated by global waves, thereby indicating supercritical behavior (Figures 6E, 7E). However, the waves were found to be more frequent and coherent under higher stimulation levels. This resulted to a large extent due to higher excitability levels, which made the cells operate in an even more ordered regime in which stochasticity is less pronounced.

**FIGURE 8:**
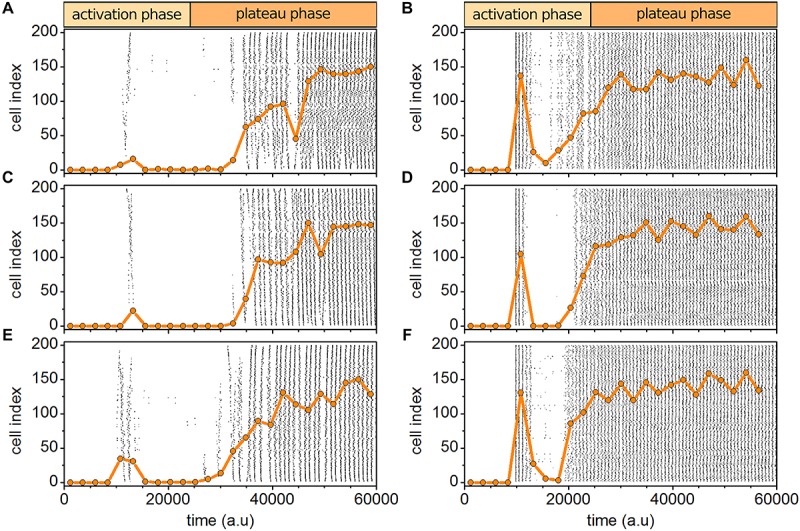
Simulated beta cell responses without particular types of cellular heterogeneities. Behavior without heterogeneity in the intrinsic excitability level **(A,B)**, without heterogeneity in the delayed responses to stimulation **(C,D)**, and without heterogeneity in intercellular coupling **(E,F)**, for both physiological **(A,C,E)** and supraphysiological **(B,D,F)** stimulation levels.

A good agreement between experimental and computational results was obtained only if all three types of heterogeneities, i.e., in excitability level, in the delayed responses to stimulation, and in intercellular coupling strengths were implemented simultaneously. To test the necessity of such a multilayered heterogeneity, we systematically performed simulations with physiological and supraphysiological stimulations without considering one of the particular heterogeneities. Results are presented in Figure 8. It can be seen qualitatively that without any of the heterogeneities the simulations do not match well with experimental results. The most obvious difference occurs in the activation phase after the initial activation, where especially in the case of physiological stimulation diverse wave sizes were observed if all three types of heterogeneities were considered. Here, on the other hand, the dynamics after the initial activation and before the system shifts to the plateau phase, is very inactive and lacks on an emergent transitory phase with progressive recruitment of cells. On the contrary, the plateau phase seems to be weakly affected by the lack of any type of variability and even if one of the heterogeneities is missing, the system behavior very similar as in control simulations. This behavior is somehow expected, since after (probably unphysiological) prolonged stimulation all cells get very excitable and placed in the supercritical regime. Also, the heterogeneity imposed by variability in metabolism diminishes. For a more quantitative evaluation we present in Table 1 the relative activity time in both phases for the experimental data, for the control simulations with 30% variability in all three types cellular heterogeneity, and for simulations without one particular aspect of heterogeneity. The results indicate that indeed the interplay between all three types of heterogeneities is necessary to firmly reproduce the experimentally observed behavior, although it seems that variability in metabolism is the most important determinant, whereas the heterogeneity in the coupling appears to be the least important.

**TABLE 1:** Activity time during physiological and supraphysiological stimulations.

	**Physiological stimulation (8** **mM glc)**			**Supraphysiological stimulation (12 mM glc)**		
	**Act. ph.**	**Plat. ph.**	**Plat./Act.**	**Act. ph.**	**Plat. ph.**	**Act./Plat.**
Exp	**3.0**	**12.0**	**4.0**	**3.9**	**16.7**	**4.3**
Sim (30% het)	**3.2**	**13.4**	**4.2**	**3.3**	**14.4**	**4.4**
No het cpl	2.0	12.6	6.3	2.1	15.3	7.3
No het exc	1.1	13.2	12.0	2.5	14.6	5.8
No het met	0.7	13.4	19.1	1.0	15.5	15.5
Het 0%	0.0	3.1	/	0.5	13.9	27.8
Het 10%	0.0	6.2	/	1.0	14.5	14.5
Het 20%	1.1	12.1	11	2.2	15.0	6.8

*Shown are percent of active time during activation phase of the response (Act. ph.), plateau phase of the response (Plat. ph.), and the relative ratio of the two (Plat./Act.), calculated for experimental data (Exp), and for the model with 30% heterogeneity in all three aspects [Sim (30% het)]. Additionally, values are depicted for the model with no heterogeneity in the coupling parameter (No het cpl), in the excitation parameter (No het exc), and in the metabolism parameter (No het met), as well as the values for no heterogeneity (Het 0%), for 10% (Het 10%), and for 20% (Het 20%) variability in all three aspects of heterogeneity. Bold values refer to original main results.*

**FIGURE 9:**
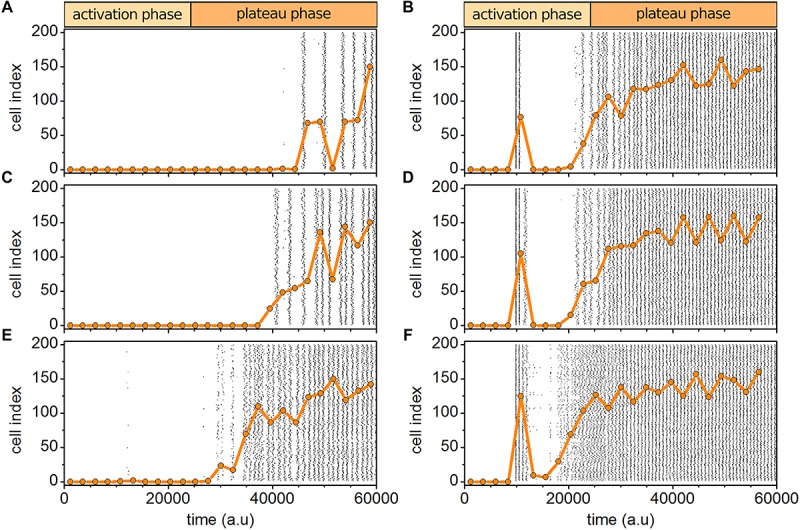
Simulated beta cell responses for different degrees of heterogeneities. Simulating the behavior without **(A,B)**, with 10% **(C,D)**, and 20% **(E,F)** of the used degree of heterogeneities for physiological **(A,C,E)** and supraphysiological **(B,D,F)** stimulation levels.

Finally, after determining that all three types of heterogeneities are required, we explored the impact of their level on the spatio-temporal activity. Figure 9 features the results. It can be observed that no or low degrees of heterogeneities fail to firmly reproduce experimental findings. In case of physiological stimulation, the initial activation of cells is missing and the cells respond much later without a progressive recruitment characterized by excitation waves of different sizes. Moreover, also the emulated supraphysiological stimulation differs if the level of heterogeneity is too low. Especially the dynamical phase after the initial activation is in this case very quiet, in contrast to the experiment and simulations with a higher degree of cellular variability, where a certain fraction of cells oscillates in this intermediate regime before the switch to the plateau phase. A quantitative assessment of this observation is presented in Table 1. It can be seen that with increasing levels of cell-to-cell variability the behavior in simulations becomes more similar to experimental results, although especially in the case with physiological stimulation 20% heterogeneity is not sufficient to achieve good consistency.

## Discussion

Information processing in living organisms is orchestrated by large networks of interacting cells. In many cases, the dynamics of these networks is guided by the activation of one or a few elements, which in turn provokes the triggering of other elements, thereby leading to avalanches of activity that propagate through the system. Such emergent behavior is associated with self-organization and very often with critical dynamics resulting in a power-law distribution of the spatial and/or temporal extent of activity profiles. This scenario is particularly appealing for excitable systems, such as neuronal networks (Plenz and Thiagarajan, 2007; Hesse and Gross, 2014; Muñoz, 2018) or excitable cells and tissues (Lopez et al., 2012; Nivala et al., 2012; Gosak et al., 2017). Typically, critical dynamics emerges at the transition between randomness (subcritical dynamics) and order (supercritical dynamics). Variations of dynamical regimes can be induced by changes of global parameters, such as the excitability level, which reflects stimulus intensity. However, in this case a power-law behavior would only be expected in a narrow parameter space in the proximity of a phase transition point. Previous research has underlined the activity-dependent synaptic plasticity, heterogeneity, and hierarchical network organization as plausible mechanisms to overwhelm this drawback. Namely, these biological determinants were found to facilitate scale-free behavior and drive neuronal networks toward the critical state (Levina et al., 2007; Rubinov et al., 2011; Moretti and Muñoz, 2013). These self-organization mechanisms make the oscillators hover around the critical point, which are therefore able to generate effective scale invariance across quite a few scales. The phenomenon is often termed as self-organized quasi-criticality (Muñoz, 2018).

In the present study, we suggest a new mechanism that realistic excitable systems might exploit for expanding the operation in a critical-like regime. As the level of cellular excitability (parameter α) increases with time, the spatio-temporal activity switches from an inactive to an active phase (Osipov et al., 2007). If excitable oscillators are homogeneous, critical behavior is expected only at the phase transition point. In our setting, where the control parameter increases with time, criticality would therefore be observed for a very short transient period of time. However, the combination of cell-to-cell variability and heterogeneously delayed increases in excitability levels substantially broaden this regime of critical-like behavior. As a result, a rather long transient dynamical phase emerges with heterogeneous wave sizes, the distribution of which closely follows a power law. Even though this critical phase is only temporary and later followed by a supercritical phase dominated by global excitatory events, critical-like behavior persists for substantial periods of time. It should be emphasized that due to the transient nature of the scale-invariant activation phase, the observed power law is only an estimation, since much higher number of events would be required to confirm a pure power-law behavior. Moreover, the present study only provides an empirical observation of transient critical-like dynamical state, and further theoretical efforts are needed to uncover the exact mechanisms for the emergence of critical dynamics in such heterogeneous systems with delayed feedbacks.

In real-life settings, it is quite common to have transient or oscillatory stimulation patterns instead of a long-lasting permanent stimulation. In neurons (Buzsaki, 2004; Schroeder and Lakatos, 2009), in pancreatic islets (Pedersen et al., 2013; Satin et al., 2015), and in cardiac myocytes (O’Rourke et al., 1994), the excitation dynamics is governed by basal variations in intrinsic excitability, for instance due to oscillations in hormone or nutrient concentrations. Notably, in our previous study we have shown that such an oscillatory entraining might be a key toward persisting criticality in pancreatic beta cells (Gosak et al., 2017). Apparently, providing proper transitory conditions for heterogeneous excitable elements that exhibit delayed and variable responses to stimulation is a viable route to scale-free behavior. Because of these heterogeneities, changeable and confined regions with elevated excitability emerge from which the excitation waves are triggered. In the activation phase, the waves are typically triggered from cells whose excitability level increased faster, whereas in the plateau phase no specific patterns can be inferred. Moreover, the range of waves in the activation phase depends on the coupling and on the variable excitable state of surrounding elements. This in turn leads to emergent behavior with very heterogeneous spatiotemporal patterns. Most importantly, the critical-like activation phase is only possible to achieve if the stimulation level is not too high. Namely, in case of supraphysiological stimulation levels, the transition to the supercritical state is too abrupt and possibly accompanied by processes that do not occur under physiological conditions, at least not to a notable extent. Consequently, excitable cells are not able to self-organize into a scale-invariant dynamical state, as is the case in physiological stimulation conditions. Cell-to-cell differences are always present to some degree in any cell population, impacting the signaling processes in various tissues and settings (Muotri and Gage, 2006; Marhl et al., 2010; Paszek et al., 2010). Notably, cellular heterogeneity is more than a nuisance and often serves a biological function or contains meaningful information (Altschuler and Wu, 2010). In islet research, beta cell heterogeneity has been one of the key issues for decades and is becoming increasingly popular, particularly in the context of subpopulations (Bader et al., 2016; Avrahami et al., 2017). Thus, the central concept of our model, i.e., multiform beta cell heterogeneity, has a long tradition. In 1987, Pipeleers defined it on grounds of structural, functional, and replicative differences between beta cells. More specifically, he pointed out differences between beta cells in terms of contact with other types of endocrine cells, in gap junctional coupling, and cellular hormone content, in their ability and sensitivity to mount a response to glucose, and in their replicative potential. Ahead of time, he argued that altered beta cell heterogeneity may turn out to be important in development of diabetes and in islet transplantation (Pipeleers, 1987). In two updates shortly thereafter, he provided evidence for functional differences between beta cells in their rates of glucose-induced insulin synthesis and secretion that were attributed to differences in thresholds for glucose utilization and oxidation. Notably, already at that time the idea was put forward that cellular heterogeneity crucially determines dose-dependence of the beta cell response to glucose due to recruitment of beta cells into an active state by increasing glucose concentrations and that elevated levels of glucose can decrease the extent of heterogeneity. Additionally, he proposed that heterogeneity is not just an experimental artifact observed in dispersed beta cells, but at work also in intact tissue (Pipeleers, 1992; Pipeleers et al., 1994). Following the advent of new molecular markers and the omics approaches, in the last decades the concept of beta cell heterogeneity has been further supported at the transcriptomic and proteomic level, together with novel findings that heterogeneity affects beta cell proliferation and survival, as well as their stimulus-secretion coupling, from glucose metabolism and Ca^2+^ signaling to insulin secretion (Benninger and Piston, 2014; Roscioni et al., 2016; Avrahami et al., 2017; Gutierrez et al., 2017; Pipeleers et al., 2017; Benninger and Hodson, 2018). Finally, it has been proposed that the lack of beta cell heterogeneity may importantly contribute to islet failure in diabetes (Johnston et al., 2016; Pipeleers et al., 2017; Skelin Klemen et al., 2017; Benninger and Hodson, 2018; Nasteska and Hodson, 2018). One major drawback, common to most recent work, is the use of dispersed beta cells. It therefore remains to be investigated to what extent the heterogeneity described thus far is translationally relevant in the tissue context or even *in vivo* (Carrano et al., 2017; Benninger and Hodson, 2018; Gosak et al., 2018; Nasteska and Hodson, 2018).

By employing the tissue slice approach, we studied a large number of coupled beta cells in their normal tissue environment. Our experimental and modeling results intersect with both the original and more recent findings on beta cell heterogeneity at several points and provide some new ideas. First, during the activation phase, differences in glucose sensitivity were observed between different cells within the same islet and these differences were larger in lower glucose (8 mM). In other words, in lower glucose, gradual recruitment of beta cells into an active state, brought about by local [Ca^2+^]_*IC*_ waves displaying critical behavior seems to be a major feature of the islet response to constant stimulation. In contrast, in high glucose (12 mM), recruitment is less well pronounced due to early global [Ca^2+^]_*IC*_ waves showing supercritical behavior. We wish to speculate that recruitment (Stožer et al., 2013a; Gosak et al., 2017) and local [Ca^2+^]_*IC*_ waves (Benninger et al., 2014; Westacott et al., 2017) in islets, as opposed to dispersed cells or clusters of cells (Jonkers and Henquin, 2001), have received less attention in the scientific community due to use of high stimulatory glucose concentrations. Second, further aspects of adaptation in the response to higher glucose are the shorter average delay to activation, shorter activation phase, and higher activity during the plateau phase. Since it has been shown recently that in addition to different pools of granules, the triggering Ca^2+^ signal importantly shapes the biphasic insulin secretion in response to constant stimulation by glucose (Pedersen et al., 2019), our findings shall importantly inform future models of beta cell insulin secretion. Additionally, the behavior of beta cells during the activation phase, when they are not functioning in synchrony with other cells, could be compared with their properties during the plateau phase to more precisely establish the relationships between their different roles. More specifically, such comparison could help answer the question whether the cells that activate first are also the ones that initiate global [Ca^2+^]_*IC*_ waves and possess the most functional connections, i.e., function as hubs, during the plateau phase (Stožer et al., 2013b; Johnston et al., 2016; Westacott et al., 2017). Third, using our phenomenological model, we found that all three types of heterogeneities, the choice of which is further substantiated in the following section, are necessary and sufficient to reproduce the experimentally observed behavior. This of course does not exclude the possibility that additional aspects of heterogeneity exist in reality and further modulate beta cell responses.

In comparison with our previous study, we used here a very simple phenomenological model to reproduce the experimentally observed non-trivial activity patterns in islets (Gosak et al., 2017). Such minimalistic modeling approaches have of course limitations, since they do not allow for any mechanistic insights into physiological processes and signaling pathways. At the same time, they offer several advantages. They are numerically very efficient, and most importantly, they contain a small number of parameters whose roles are rather clear, which makes it easier to explicitly study particular aspects of cellular heterogeneity. In contrast, realistic and multi-component cellular models exhibit many parameters that in general affect several aspects of signalization, which hinders a systematic and definitive examination of their particular influences on cellular behavior. Finally, it should be noted that the majority of existing comprehensive beta cell models were mainly focused on the activity on the plateau phase, whereas modeling of collective cellular activations after switching from substimulatory to (supra)stimulatory glucose received very little attention. The mechanisms that govern such stimulus-dependent activation are also understood incompletely. Phenomenological modeling is therefore beneficial in this respect, as long as the empirical description of the processes ensures good agreement between modeling and experimental results. In particular, the time lags and temporal evolution of the excitability level [see eq. (3)] are plausible processes that can be qualitatively linked with previous experimental observations, such as differences in metabolic sensitivity to glucose and the following electrical and [Ca^2+^]_*IC*_ responses (Stožer et al., 2013a,b; Benninger et al., 2014; Farnsworth and Benninger, 2014; Johnston et al., 2016). Moreover, we decided to include heterogeneity in intercellular coupling due to the extensive experimental support demonstrating its importance in both normal and pathological islet functioning (Hodson et al., 2013; Farnsworth et al., 2014, 2016; Johnston et al., 2016; Skelin Klemen et al., 2017). However, here we focused only on the fast [Ca^2+^]_*IC*_ oscillations and future studies will conceivably need to include additional aspects of heterogeneity to provide a comprehensive and realistic beta cell model capable of describing other components of the [Ca^2+^]_*IC*_ pattern, other parameters in the stimulus-secretion cascade, as well as responses to different levels of stimulation and different secretagogues.

Multiscale and multidimensional heterogeneity represent a viable route to critical-like behavior for a substantial period of time. Specifically, the dynamical transition between the inactive and active state occurs in a rather broad temporal interval, especially in the case of physiological levels of stimulation. In other words, as the glucose level increases the activation of cells is not abrupt. Before switching to the dynamical state with global Ca^2+^ events, a transient period of very heterogeneous wave sizes is observed, which implies a critical-like behavior, since the system as a whole bypasses the critical point rather slowly. On the other hand, supraphysiological high stimulation levels lack on such progressive recruitment of cells and lead to a rapid transition to a fully active state. This might be crucial for healthy physiological functioning of pancreatic islets and potentially of other biological tissues as well. Namely, for other biological tissues, there is a large body of evidence indicating severe pathophysiological consequences of an abrupt collective transitions to hyper-regulated synchronous tissue responses. An overview was given by Trefois et al. (2015), showing that critical transitions are identified as early warning signals for the onset of different pathologies ranging from microbiome dysregulations to irritable bowel syndrome, asthma, pulmonary disease, depression, type 1 and type 2 diabetes, inflammation, start and termination of epileptic seizures, cancer, and cardiovascular events.

Further investigations are needed to understand the onset of pathological supercritical behavior in more detail. The molecular and cellular mechanisms are still obscure; however, the results of our study, although only empirical, give at least a hint to an improved methodology, opening a new dimension in studying the (premature) onset of supercriticality by looking at the extent of cell heterogeneity. Some preliminary studies in our lab show that beta cell responses in terms of [Ca^2+^]_*IC*_ signals shall also be correlated with other aspects of heterogeneity to get a more complete picture about the mechanisms that make some cells more responsive to glucose and to find out whether this is a stable property or something that changes with time and on which temporal scale. In addition, different concentrations of glucose and additional stimulation protocols, as well other secretagogues shall be used in future studies. Moreover, the general extent of heterogeneity in the islets, as well as the properties of individual cells shall be investigated in mouse models of diabetes and in human islets from normal and diabetic donors to more clearly define the changes under pathological conditions and suggest targets for treatment (Benninger and Hodson, 2018; Stožer et al., 2019). In general, from the viewpoint of clinical approaches, the understanding of critical transitions might help us develop therapies that are more effective. From the viewpoint of preventive health care, an improved understanding of the pathological premature transitions to supercriticality could help us identify and characterize some early warning signals predicting the upcoming pathological transitions. Finally, beyond the preventive and therapeutic role Bargaje et al. (2017) showed that an increase in cell heterogeneity of stem cells just before the critical transition correlates with a branching point on the trajectory of cell fate. This represents a useful tool for forecasting the cell fate outcomes and can be used for optimizing the differentiation protocols in order to obtain desired cell populations, which opens a completely new dimension of bioengineering in the future.

## Data Availability

The datasets generated for this study are available on request to the corresponding author.

## Ethics Statement

The study was conducted in strict accordance with all national and the European recommendations pertaining to care and work with experimental animals, and all efforts were made to minimize suffering of animals. The protocol was approved by the Veterinary Administration of the Republic of Slovenia (Permit Number: U34401-12/2015/3).

## Author Contributions

AS, RM, JD, MP, MSR, MM, and MG designed the study, carried out the research, and wrote the manuscript.

## Conflict of Interest Statement

The authors declare that the research was conducted in the absence of any commercial or financial relationships that could be construed as a potential conflict of interest.
